# Promoter Methylation and Somatic Mutations in Cancer-Related Genes Are Associated with Hyperprogressive Disease in Patients with Malignant Melanoma and Renal Cell Carcinoma Receiving Anti-PD-1/PD-L1 Immunotherapy

**DOI:** 10.3390/jcm15135089

**Published:** 2026-06-30

**Authors:** Adem Deligonul, Mehmet Sarimahmut, Ahmet Bilgehan Sahin, Elif Erturk, Engin Atli, Hazal Sezginer Guler, Erdem Cubukcu, Hulya Ozturk Nazlioglu, Saduman Balaban Adim, Turkkan Evrensel, Ferda Ari

**Affiliations:** 1 Department of Medical Oncology, School of Medicine, Bursa Uludag University, 16059 Bursa, Turkey; ademd@uludag.edu.tr (A.D.); absahin@uludag.edu.tr (A.B.S.); erdemcubukcu@uludag.edu.tr (E.C.); evrensel@uludag.edu.tr (T.E.); 2 Department of Biology, Faculty of Science and Arts, Bursa Uludag University, 16059 Bursa, Turkey; msarimahmut@uludag.edu.tr; 3 Vocational School of Health Services, Bursa Uludag University, 16059 Bursa, Turkey; eliferturk@uludag.edu.tr; 4 Department of Medical Genetics, Faculty of Medicine, Trakya University, 22030 Edirne, Turkey; enginatli@trakya.edu.tr (E.A.); hazalsezginer@trakya.edu.tr (H.S.G.); 5 Department of Pathology, School of Medicine, Bursa Uludag University, 16059 Bursa, Turkey; hulyaozturk@uludag.edu.tr (H.O.N.); balabanadim@uludag.edu.tr (S.B.A.)

**Keywords:** hyperprogressive disease, immune checkpoint inhibitors, promoter methylation, next-generation sequencing, malignant melanoma, renal cell carcinoma

## Abstract

**Background and Objectives:** A subset of cancer patients treated with immune checkpoint inhibitors may experience rapid tumor progression rather than therapeutic benefit, a phenomenon described as hyperprogressive disease (HPD), which is linked to poor prognosis and shortened survival. Reliable biomarkers capable of predicting HPD remain lacking. To better understand the molecular background of HPD, we analyzed promoter region methylation and somatic mutation profiles in cancer-related genes in patients with malignant melanoma (MM) and renal cell carcinoma (RCC) undergoing anti-PD-1/PD-L1 treatment. **Methods:** Patients diagnosed with MM or RCC and treated with anti-PD-1/PD-L1 agents between 2011 and 2020 were included, and FFPE tumor samples along with paired normal tissues were analyzed. A diagnosis of HPD was assigned to patients with RECIST 1.1-defined progressive disease who demonstrated a ≥2-fold acceleration in tumor growth kinetics after initiation of immune checkpoint inhibitor therapy. Methylation-specific real-time PCR was performed on 54 samples (15 MM tumors, 22 RCC tumors, 17 RCC-matched adjacent normal samples) to assess promoter methylation of *PIK3CA*, *BAP1*, *PTEN*, and *TP53*. Next-generation sequencing (NGS) with an 86-gene pan-cancer panel was conducted on 9 HPD samples. **Results:** Promoter hypermethylation involving *PIK3CA, BAP1, PTEN*, and *TP53* was more pronounced in HPD-associated tumor samples (*n* = 16) than in tumors without HPD (*n* = 21). Within the MM cohort, *PTEN* and *TP53* methylation levels demonstrated statistically significant differences between the two groups (*p* = 0.005 and *p* = 0.028, respectively), while no comparable associations were observed in RCC patients. NGS analysis detected missense mutations classified as pathogenic or likely pathogenic in 5 of 9 HPD patients (55.6%), involving *KIT*, *PTEN*, and *VHL*. **Conclusions:** Promoter region hypermethylation in cancer-related genes may contribute to the aggressive tumor behavior observed in HPD. The somatic variants identified in HPD patients are consistent with known oncogenic pathways. These findings support further investigation of epigenetic and genomic biomarkers for HPD risk stratification in larger, prospective cohorts.

## 1. Introduction

Therapies targeting the PD-1/PD-L1 immune checkpoint axis have become an important component of treatment for several solid malignancies, including MM [1] and RCC [2]. Despite favorable and long-lasting responses in some individuals, certain patients exhibit HPD, which is defined by accelerated tumor progression following the initiation of ICI (immune checkpoint inhibitor) treatment [3,4]. HPD is clinically distinct from pseudoprogression, in which radiological enlargement reflects immune cell infiltration rather than true disease progression; in HPD, tumor growth kinetics accelerate and the patient’s clinical condition deteriorates [4].

The occurrence of HPD has been documented in several malignancies undergoing PD-1/PD-L1 blockade therapy, with reported frequencies ranging from 1% to 30% based on tumor subtype, patient characteristics, and diagnostic definitions. Previous studies have estimated HPD rates of 6–10% in MM and 1–7% in RCC [5]. Moreover, patients developing HPD generally exhibit worse clinical outcomes, including shorter overall and progression-free survival, compared with patients showing conventional progression patterns [6]. However, a standardized definition of HPD remains absent. Several parameters, including tumor growth rate, tumor growth kinetics (TGK), and time to treatment failure, have been proposed for the evaluation of HPD. Nevertheless, the lack of standardized criteria across research groups has prevented their incorporation into established response evaluation frameworks such as RECIST 1.1, iRECIST, and imRECIST [7]. This lack of consensus complicates cross-study comparisons and limits the identification of reliable predictive biomarkers.

The molecular mechanisms underlying HPD are poorly understood [8]. Limited evidence suggests that somatic mutations, gene amplifications (particularly *MDM2*, *MDM4,* and *EGFR*) [9] as well as epigenetic alterations [10] may contribute to the accelerated tumor growth observed after ICI exposure. Methylation-based biomarker studies in RCC are scarce, and in MM the relationship between promoter hypermethylation and HPD has not been directly assessed. Identifying molecular correlates of HPD is therefore critical to stratify patient risk prior to ICI initiation and to elucidate the biological underpinnings of this phenomenon [11,12,13].

Promoter methylation status of *PIK3CA, BAP1, PTEN,* and *TP53*, as well as somatic mutation profiles, were examined in MM and RCC patients stratified according to HPD status following anti-PD-1/PD-L1 therapy. Methylation-specific real-time PCR together with targeted NGS was applied to identify genomic and epigenetic alterations that may contribute to HPD development.

## 2. Materials and Methods

### 2.1. Study Population and Sample Collection

A retrospective cohort of MM and RCC patients receiving anti-PD-1/PD-L1 immunotherapy at Bursa Uludag University Hospital between 2011 and 2020 was included in the study. Patients aged > 18 years with ECOG (Eastern Cooperative Oncology Group) performance status 0–2 and stage IV RCC or MM were eligible for inclusion. All MM cases included in the study were histopathologically classified as cutaneous melanoma, and all RCC cases classified as HPD were clear cell RCC. All patients were required to have at least 12 weeks of follow-up after immunotherapy initiation and to have undergone radiological and clinical response assessment within 6–8 weeks after treatment initiation. In RCC, immunotherapy was generally administered after prior systemic therapy, most commonly following tyrosine kinase inhibitor treatment. Patients receiving adjuvant immunotherapy, patients without 6–8-week imaging or accessible clinical data, and patients whose archival paraffin blocks were unavailable were excluded. HPD classification was therefore performed using a complete-case approach, including only patients with sufficient clinical and radiological data for pre-treatment and post-treatment tumor growth assessment.

**Figure 1:**
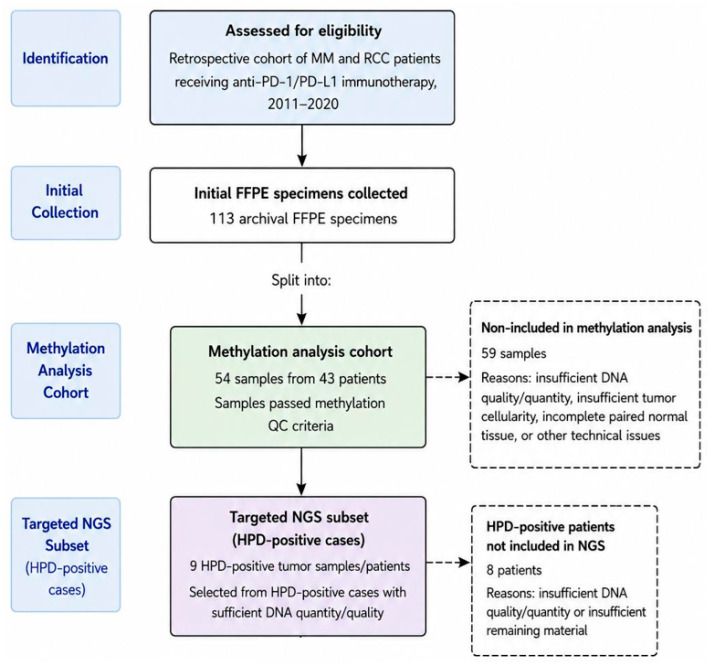
CONSORT-style flow diagram illustrating patient selection, archival FFPE specimen availability, methylation analysis eligibility, targeted NGS subset selection, and non-included samples/patients in patients with malignant melanoma (MM) and renal cell carcinoma (RCC) treated with anti-PD-1/PD-L1 immunotherapy.

Tumor FFPE samples and paired normal tissues totaling 113 specimens were collected from the Department of Pathology archives. Molecular analyses were performed on samples that met the corresponding DNA quantity and quality requirements for methylation-specific real-time PCR or targeted NGS. Targeted NGS was restricted to selected HPD-positive tumor samples with sufficient DNA quantity and quality for sequencing. A CONSORT-style flow diagram summarizing patient selection, FFPE tissue availability, quality-control filtering, methylation analysis eligibility, and targeted NGS subset selection is provided in Figure 1. Available sample-level and patient-level comparisons of included and non-included cases are provided in Supplementary Tables S1 and S2.

Ethical approval for the study was granted by the Bursa Uludag University Hospital Research Ethics Committee (approval no. 2019-20/24), and the study was carried out in accordance with the Declaration of Helsinki.

### 2.2. HPD Definition

Radiological response was evaluated according to RECIST 1.1. Target lesion diameters were assessed on the most recent available pre-baseline scan, the baseline scan obtained before initiation of anti-PD-1/PD-L1 therapy, and the first response-assessment scan performed 6–8 weeks after treatment initiation. TGK was calculated using the linear sum of measurable target lesion diameters divided by the time interval between radiological assessments. Pre-treatment TGK was calculated as

TGK_pre_ = (SLD_baseline_ − SLD_pre-baseline_)/Δt_pre-baseline−baseline_

and post-treatment TGK was calculated as

TGK_post_ = (SLD_post-treatment_ − SLD_baseline_)/Δt_baseline−post-treatment_

where SLD represents the sum of target lesion diameters and Δt represents the time interval between scans. Patients meeting RECIST 1.1 criteria for progressive disease after initiation of anti-PD-1/PD-L1 therapy were classified as having HPD if the TGKpost/TGKpre ratio demonstrated a twofold or greater increase [7]. Progression occurring within 2 months of ICI initiation with concurrent clinical deterioration or worsening ECOG performance status was also required [14]. New lesions were considered according to RECIST 1.1 for the determination of progressive disease, whereas TGK calculations were based on measurable target lesion diameters. A limitation of this retrospective approach is that TGK was derived from linear target lesion diameter measurements, and volumetric or exponential tumor growth modeling could not be performed.

### 2.3. DNA Isolation and Quality Assessment

FFPE tumor specimens and paired normal tissue sections were processed for genomic DNA isolation using commercially available kits from Qiagen (Hilden, Germany) and Omega Bio-tek (Norcross, GA, USA). DNA yield and purity were determined using a NanoDrop 2000 spectrophotometer (Thermo Fisher Scientific, Waltham, MA, USA). After the extraction procedure, genomic DNA was stored at −20 °C for subsequent analyses.

### 2.4. Methylation-Specific Real-Time PCR

The EZ DNA Methylation-Gold Kit (Zymo Research, Irvine, CA, USA) was used for bisulfite treatment of genomic DNA according to the manufacturer’s protocol. Briefly, genomic DNA was incubated with CT Conversion Reagent using the following thermal profile: 98 °C for 10 min, 64 °C for 2.5 h, and 4 °C hold, followed by column-based desulphonation, washing, and elution. Following conversion, promoter region methylation of *PIK3CA*, *BAP1*, *PTEN*, and *TP53* was examined by MS-RT-PCR. CpG island-specific methylated and unmethylated primer pairs were designed using MethPrimer 2.0 software (Ractigen Therapeutics, Shenzhen, China) based on NCBI and Ensembl reference sequences. Full MS-RT-PCR primer and amplicon details, including reference sequences, methylated/unmethylated primer sequences, product sizes, predicted melting temperatures, and MethPrimer-reported start positions, are provided in Supplementary Table S3. MethPrimer CpG island and primer-position outputs are shown in Supplementary Figure S1.

MS-RT-PCR was performed on a LightCycler 480 II instrument (Roche, Mannheim, Germany) using LightCycler 480 SYBR Green I Master chemistry (Roche, Mannheim, Germany). Each 10 µL reaction contained 5.0 µL SYBR Green I Master mix, 0.3 µL forward primer, 0.3 µL reverse primer, 1.9 µL PCR-grade water, and 2.5 µL bisulfite-converted DNA. The amplification protocol consisted of pre-incubation at 95 °C for 5 min, followed by 45 cycles of 95 °C for 10 s, 59 °C for 10 s, and 72 °C for 10 s. Melting-curve analysis was performed at 95 °C for 5 s and 64 °C for 1 min, followed by continuous acquisition up to 97 °C. Cooling was performed at 40 °C for 30 s.

In this MS-RT-PCR workflow, methylation status was confirmed using positive methylated control DNA. Samples showing only unmethylated-specific melting signals were reported as 0% methylation, samples showing only methylated-specific melting signals were reported as 100% methylation, and samples showing both methylated and unmethylated signals were assigned intermediate methylation percentages based on melting-curve peak-height fluorescence values and standard-curve calibration. The distribution of 0%, intermediate, and 100% methylation values for each gene is summarized in Supplementary Table S4.

### 2.5. Next-Generation Sequencing

Nine HPD samples that passed quality control were analyzed using the KAPA HyperPETE Pan-Cancer Panel (Roche, Basel, Switzerland), targeting 86 cancer-related genes (Supplementary Table S5). Sequencing data were generated using the MGI DNBSEQ-400 system (MGI Tech, Shenzhen, China). Bioinformatic processing of raw data was performed with CLC Genomics Workbench version 25.2 and downstream variant interpretation utilized Qiagen Clinical Insight (QCI) software version 9.2.3. All detected variants were additionally examined in IGV (Broad Institute) for visual confirmation. Variant pathogenicity was evaluated using multiple public and clinical databases, including HGMD, NCBI, dbSNP, VarSome, and Franklin (Genoox).

The clinical relevance of variants meeting the defined thresholds was evaluated using the AMP/ASCO/CAP consensus framework [15], which classifies variants into Tier I–IV categories based on their level of clinical significance. Targeted NGS was performed as an exploratory analysis in selected HPD-positive archival pre-immunotherapy FFPE tumor samples that met DNA quantity and quality requirements for sequencing.

### 2.6. Statistical Analysis

Promoter methylation percentages for *PIK3CA*, *BAP1*, *PTEN*, and *TP53* were compared between HPD and non-HPD groups separately for MM and RCC tumor samples using independent-samples *t*-tests. In the RCC subgroup, paired tumor and adjacent normal tissues were available for analysis, enabling paired *t*-test comparisons of methylation levels according to HPD status. Because methylation percentage data are bounded variables and may include low or zero values, the assumptions underlying parametric testing may not be fully satisfied; therefore, the statistical findings should be interpreted within the exploratory framework of the study. Given the exploratory framework of the study together with the limited sample number, no multiple-testing correction was implemented, and the findings should therefore be evaluated cautiously. Graphical representations were generated using GraphPad Prism 9.1.2 (GraphPad Software, San Diego, CA, USA), whereas all statistical analyses were performed in SPSS version 29.0.2.0 (IBM, Armonk, NY, USA). A two-sided *p*-value below 0.05 was accepted as statistically significant.

Kaplan–Meier survival analysis was performed using SPSS version 29.0.2.0 among HPD-positive patients to visualize overall survival from the initiation of anti-PD-1/PD-L1 immunotherapy according to tumor type (MM versus RCC). Overall survival was defined as the time from the start of immunotherapy to death. Survival distributions were compared using the log-rank test.

## 3. Results

### 3.1. Methylation-Specific RT-PCR Analysis

Of the 113 FFPE samples collected, 54 met quality criteria for methylation analysis. These comprised 15 MM tumor samples (6 HPD, 9 non-HPD), 22 RCC tumor samples (10 HPD, 12 non-HPD), and 17 RCC matched normal tissue samples (5 HPD, 12 non-HPD). Individual methylation percentages for all samples are reported in Table 1.

Elevated promoter methylation of *PIK3CA*, *BAP1*, *PTEN*, and *TP53* was detected in HPD-associated tumors (*n* = 16) relative to non-HPD tumors (*n* = 21) in the combined cohort. Stratified analyses revealed statistically significant differences for *PTEN* and *TP53* methylation in MM patients (*p* = 0.005 and *p* = 0.028, respectively), whereas no comparable gene-level associations were identified in RCC samples.

**Figure 2:**
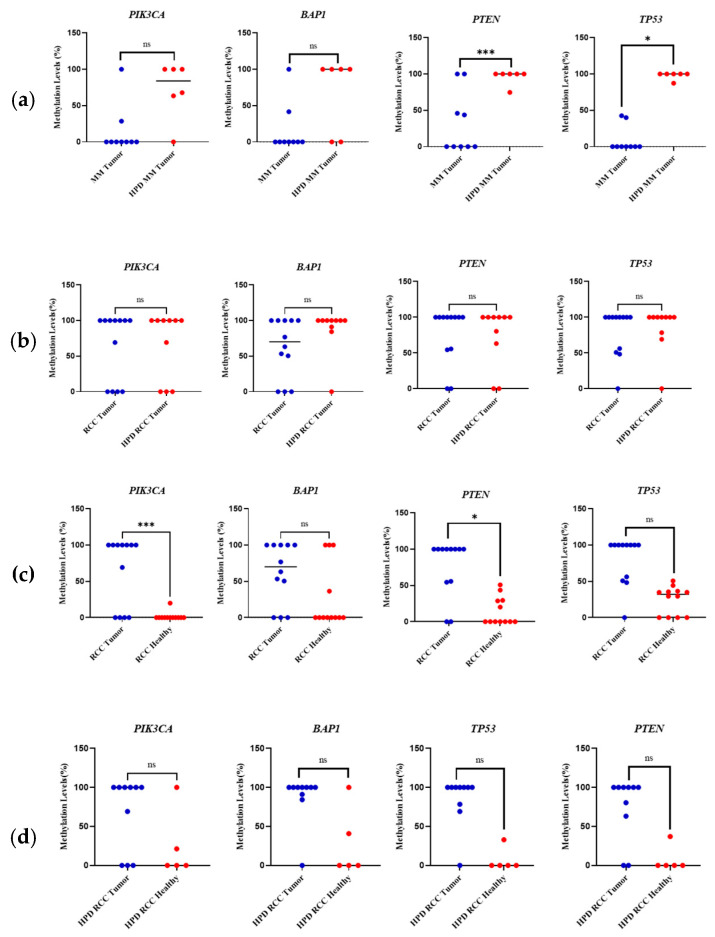
Promoter Region Methylation Levels of *PIK3CA, BAP1, PTEN*, and *TP53* in HPD and Non-HPD Tumor Samples. Comparisons were performed using independent-samples *t*-tests, (**a**). Comparison between MM HPD tumor samples and MM non-HPD tumor samples, (**b**). Comparison between RCC HPD tumor samples and RCC non-HPD tumor samples, (**c**). Comparison between RCC healthy (matched normal) tissues and RCC tumor tissues, (**d**). Comparison between RCC HPD tumor samples and matched RCC healthy tissues. Statistically significant differences are indicated with asterisks (* *p* < 0.05, *** *p* = 0.005).

In the RCC subset, paired comparison of tumor and matched normal tissue revealed no significant differences in methylation levels among HPD patients. Non-HPD RCC cases exhibited higher methylation levels in tumor samples than in matched adjacent normal tissues for both *PIK3CA* and *PTEN*, reaching statistical significance (*p* < 0.001 and *p* = 0.024, respectively). These methylation patterns are illustrated in Figure 2.

### 3.2. Next-Generation Sequencing

Nine HPD patients (5 MM, 4 RCC) who passed sequencing quality control were included in NGS analysis. No pathogenic variants were detected in one RCC sample (patient 55). In the remaining eight patients, 13 distinct variants were identified—5 classified as P (pathogenic) or LP (likely pathogenic) and 8 as variants of VOUS (uncertain significance). All detected variants were missense mutations resulting in amino acid substitutions. Detailed variant information is provided in Table 2.

Among patients with MM, a likely pathogenic *KIT* variant (NM_000222.3:c.1924A>G, p.Lys642Glu; allele fraction 43%) was detected in patient 49, and a pathogenic *PTEN* variant (NM_000314.8:c.988A>G, p.Lys330Glu; allele fraction 48%) was identified in patient 50. Among patients with RCC, a likely pathogenic *VHL* variant (NM_000551.4:c.344A>C, p.His115Pro; allele fraction 26%) was found in patient 57, a pathogenic *KIT* variant (NM_000222.3:c.1924A>C, p.Lys642Gln; allele fraction 38%) in patient 59, and a pathogenic *VHL* variant (NM_000551.4:c.332G>A, p.Ser111Asn; allele fraction 29%) in patient 61. To provide additional biological context for the exploratory genomic findings, publicly available TCGA PanCancer Atlas datasets were explored through the cBioPortal platform. OncoPrint analyses of *PTEN, TP53, PIK3CA, BAP1, KIT*, and *VHL* were generated using the TCGA Skin Cutaneous Melanoma (SKCM) and Kidney Renal Clear Cell Carcinoma (KIRC) cohorts and are presented in Supplementary Figure S2.

### 3.3. Survival Analysis

**Figure 3:**
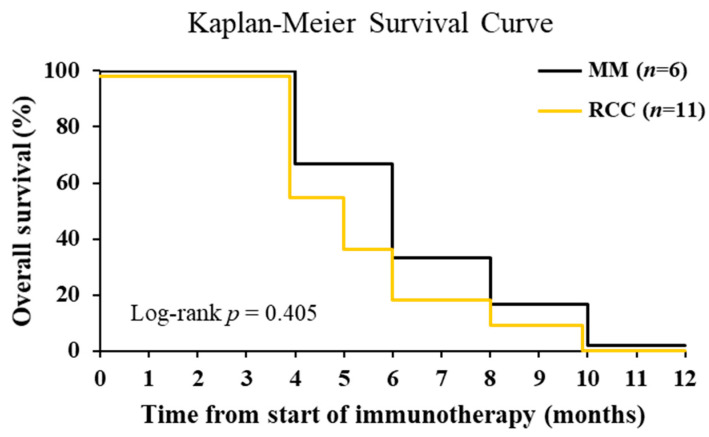
Kaplan–Meier analysis of overall survival from initiation of anti-PD-1/PD-L1 immunotherapy among HPD-positive patients according to tumor type. Survival curves were compared using the log-rank test (*p* = 0.405). Abbreviations: MM, malignant melanoma; RCC, renal cell carcinoma.

Kaplan–Meier analysis was performed in HPD-positive patients to compare overall survival from the initiation of anti-PD-1/PD-L1 immunotherapy between MM and RCC cases. The analysis included 17 HPD-positive patients with available survival data, comprising 6 MM and 11 RCC patients. The difference between the survival curves was not statistically significant by log-rank test (*p* = 0.405) (Figure 3).

### 3.4. Integrated Clinical and Molecular Characterization of the Cohort

**Figure 4:**
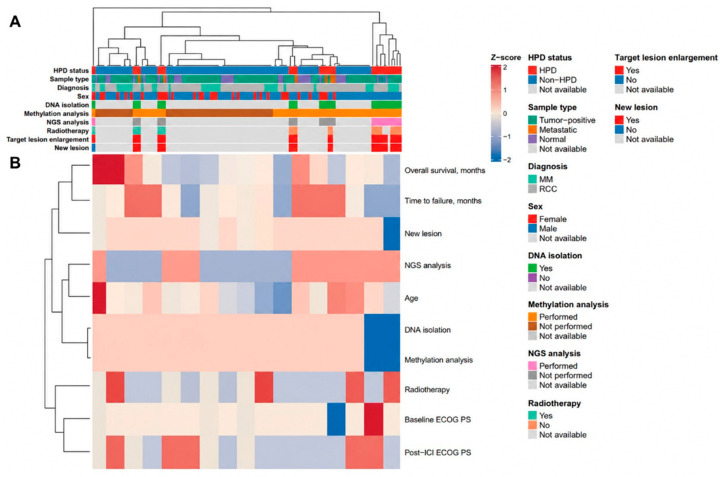
Integrated clinicopathological and molecular-analysis status heatmap of the study cohort. (**A**) Unsupervised hierarchical clustering of the overall study cohort based on available clinical, radiological, sample-processing, and molecular-analysis availability. (**B**) Integrated heatmap restricted to HPD-positive patients, incorporating available outcome- and progression-related variables. Red and blue colors indicate relatively higher and lower scaled values, respectively, whereas grey indicates unavailable or non-applicable data. Categorical annotation colors are defined in the legend. Abbreviations: HPD, hyperprogressive disease; ICI, immune checkpoint inhibitor; MM, malignant melanoma; RCC, renal cell carcinoma; NGS, next-generation sequencing; ECOG PS, Eastern Cooperative Oncology Group performance status.

An integrated heatmap based on unsupervised hierarchical clustering was generated to visualize clinical and molecular characteristics across the study cohort (Figure 4). The analysis incorporated HPD status, clinical parameters, promoter methylation profiles, and targeted NGS findings, providing a comprehensive overview of patient-level heterogeneity.

## 4. Discussion

Our analysis of promoter methylation and somatic mutation profiles in MM and RCC patients treated with anti-PD-1/PD-L1 immunotherapy identified distinct epigenetic alterations associated with HPD. In particular, MM patients with HPD demonstrated increased *PTEN* and *TP53* promoter hypermethylation, while RCC patients showed no significant gene-level methylation changes related to HPD status. In parallel, NGS analysis identified pathogenic or likely pathogenic variants in *KIT*, *PTEN*, and *VHL* in 5 of 9 HPD patients, suggesting that both epigenetic and genomic alterations may coexist in tumors with hyperprogressive behavior.

HPD rates in the methylation subset—40% for MM (6/15) and 45.5% for RCC (10/22)—were substantially higher than the ranges commonly reported in the literature, which has been observed at variable frequencies, generally estimated at 1–30% across solid cancers undergoing PD-1/PD-L1 blockade therapy [5]. These proportions should not be interpreted as population-level HPD incidence estimates, because the methylation cohort was restricted to patients with available FFPE tumor samples meeting DNA quality requirements for molecular analysis. Several factors may therefore account for this discrepancy, including the limited sample size, the impact of individual patient categorization on subgroup proportions, and potential enrichment related to tissue availability and molecular-analysis eligibility. In addition, the RCC subgroup represented a clinically aggressive population, with patients classified within intermediate or poor IMDC prognostic groups. It should be noted that published HPD incidence estimates also vary widely depending on the diagnostic criteria adopted—a factor that further complicates direct comparisons across studies [5,16]. Establishing accurate HPD incidence rates in MM and RCC specifically will require larger, prospective, multi-center cohorts.

From a biological standpoint, the association between *PTEN* and *TP53* promoter hypermethylation and HPD in MM is plausible and consistent with known oncogenic mechanisms. *PTEN* loss—whether through mutation, deletion, or epigenetic silencing—activates the PI3K/AKT/mTOR signaling axis, promoting cell survival and proliferation [17,18]. When *PTEN* promoter hypermethylation is associated with reduced *PTEN* expression, this inhibitory brake on *PI3K* signaling is removed, potentially facilitating rapid tumor expansion in the context of immune checkpoint modulation [19]. Similarly, *TP53* promoter hypermethylation or reduced *TP53* function may impair apoptotic and cell-cycle checkpoint responses, which may allow tumor cells to evade growth control under immune selective pressure [20]. The co-occurrence of hypermethylation in both genes within the same HPD samples raises the possibility that coordinated epigenetic dysregulation of multiple tumor suppressor genes—including *BAP1*, *PTEN* and *TP53*—can create a permissive molecular environment for hyperprogression—a hypothesis that merits functional validation. Although increased *PTEN* and *TP53* promoter hypermethylation was observed in patients with hyperprogressive disease, functional validation analyses such as RNA expression profiling, immunohistochemistry, or protein-level assessment were not performed in the present study. Therefore, these findings should be interpreted cautiously and not considered definitive evidence of transcriptional silencing.

In RCC, the absence of significant gene-level methylation differences between HPD and non-HPD tumor samples may reflect the distinct epigenetic and genomic landscape of this malignancy. Clear cell RCC is characterized by frequent *VHL* inactivation and mutations in genes associated with chromatin remodeling pathways, including *PBRM1*, *SETD2*, and *BAP1*, which operate through mechanisms that differ from classical promoter hypermethylation of tumor suppressors [21]. In this context, it is noteworthy that non-HPD RCC patients showed significant tumor-versus-normal methylation differences for *PIK3CA* and *PTEN*, whereas HPD RCC patients did not. One possible interpretation is that the absence of differential methylation between tumor and normal tissue in HPD patients reflects a broader epigenetic dysregulation extending beyond the tumor itself—potentially a field effect or systemic epigenetic predisposition. However, this observation is based on a limited number of matched normal samples (*n* = 5 for HPD) and remains preliminary.

The NGS findings, although derived from a small cohort, provide additional molecular context. Among the pathogenic and likely pathogenic variants, two distinct *KIT* exon 13 variants were detected—p.Lys642Glu in patient 49 (MM) and p.Lys642Gln in patient 59 (RCC). Both residues map to the tyrosine kinase domain, and mutations involving KIT exons 9, 11, 13, and 17 have previously been correlated with imatinib responsiveness [22]. *KIT*-mutant melanoma is a recognized molecular subtype with distinct therapeutic implications [23]. Whether *KIT* mutations also influence the likelihood of HPD during immunotherapy is an open question that warrants dedicated investigation. The *PTEN* pathogenic variant detected in MM patient 50 is functionally consistent with the methylation findings, given that *PTEN* participates in *MDM2* pathway regulation [24]—a pathway directly implicated in HPD pathogenesis through *MDM2*/*MDM4* amplification [9]. The *VHL* variants identified in RCC patients 57 and 61 are in accordance with the established role of *VHL* as the most frequently mutated gene in clear cell RCC [25], although their specific contribution to hyperprogressive behavior remains to be determined.

Survival analysis further demonstrated the poor clinical course of HPD-positive patients in this cohort. Median overall survival from immunotherapy initiation was short in both tumor types, with no statistically significant difference between MM and RCC patients.

The findings of this study should be interpreted in light of several important limitations. First, the sample size was small, particularly for the NGS analysis (*n* = 9), which limited statistical power and precluded multivariable analyses. Second, the retrospective single-center design may introduce potential selection and information biases. In addition, formal adjustment for baseline disease aggressiveness could not be reliably performed because of the limited cohort size and incomplete availability of LDH, baseline tumor burden, and metastatic-site data. Although RCC patients were classified within intermediate or poor IMDC (International Metastatic RCC Database Consortium) prognostic groups, this does not substitute for multivariable adjustment. Therefore, the observed methylation associations should be interpreted as exploratory rather than adjusted biomarker associations. Third, no correction for multiple comparisons was applied across the four genes tested in the methylation analysis; the reported *p*-values should therefore be interpreted as exploratory. Fourth, matched normal tissue was available only for RCC patients, preventing equivalent tumor-versus-normal comparisons in MM. In addition, melanoma subtype-specific analyses (e.g., cutaneous versus mucosal melanoma) could not be performed because of the limited number of available cases, despite the recognized biological heterogeneity between these subtypes. Fifth, external validation in an independent cohort was not performed. Finally, the methylation analysis was limited to four candidate genes selected a priori. In this scenario, genome-wide methylation profiling—such as array-based or whole-genome bisulfite sequencing approaches—could reveal additional epigenetic markers associated with HPD. Similarly, the NGS findings remain descriptive because sequencing was restricted to a small HPD-positive subset without a non-HPD sequencing comparator; therefore, the detected variants should be regarded as hypothesis-generating rather than HPD-specific molecular associations.

Although a Kaplan–Meier analysis was added to visualize overall survival among HPD-positive patients, the current cohort size and retrospective design do not permit the development of a statistically reliable predictive scoring system or survival-based risk model. Future prospective multicenter studies integrating molecular, clinicopathological, and survival data will be essential to establish clinically actionable biomarkers capable of predicting HPD risk before initiation of immune checkpoint inhibitor therapy.

Although several limitations should be acknowledged, our study provides one of the first comparative evaluations of promoter methylation status in relation to HPD development in MM and RCC. The convergence of promoter hypermethylation and somatic mutations in tumor suppressor genes within HPD samples supports the hypothesis that multiple, potentially synergistic molecular mechanisms may underlie the aggressive tumor behavior observed in hyperprogressive patients. Future studies integrating methylation profiling, mutational analysis, and transcriptomic data in larger, prospective cohorts will be essential to validate these preliminary findings and to move toward clinically actionable HPD risk stratification tools. Future studies should incorporate integrated multidimensional approaches combining clinical variables, tumor subtype information, methylation profiles, genomic alterations, and survival outcomes to better characterize the complex molecular background of HPD.

## 5. Conclusions

Promoter region hypermethylation of *PTEN* and *TP53* was significantly associated with HPD in MM patients, while no gene-level differences were observed in RCC. NGS analysis identified pathogenic or likely pathogenic missense variants in *KIT*, *PTEN*, and *VHL* in 5 of 9 HPD patients. These preliminary findings indicate that promoter hypermethylation of tumor suppressor genes, together with observed somatic alterations, may be associated with aggressive tumor behavior observed in patients with hyperprogressive disease. Validation in larger, prospective, multi-center cohorts is warranted to determine whether these molecular markers can be integrated into clinical HPD risk stratification.

## Figures and Tables

**Table 1:** Promoter Region Methylation Percentages of *PIK3CA*, *BAP1*, *PTEN*, and *TP53* in Tumor and Matched Normal Tissue Samples, Stratified by Cancer Type and HPD Status.

Sample Group	Sample No.	*PIK3CA* (%)	*BAP1* (%)	*PTEN* (%)	*TP53* (%)
**MM tumor—non-HPD (*n* = 9)**	10	0	41.53	45.90	0
	16	0	100	100	0
	17	100	0	0	0
	18	0	0	43.72	0
	19	0	0	0	0
	22	0	0	100	0
	25	0	0	0	42.56
	26	28.52	0	0	0
	27	0	0	0	39.90
**MM tumor** **—** **HPD (*n* = 6)**	49	100	0	100	100
	50	100	0	100	100
	51	67.78	100	100	100
	52	0	100	74.69	100
	53	100	100	100	100
	54	63.39	100	100	87.29
**RCC tumor** **—** **non-HPD (*n* = 12)**	29	0	0	54.45	0
	30	0	100	100	100
	31	100	100	100	48.36
	33	100	100	55.67	100
	34	100	53.32	0	100
	35	100	0	100	100
	39	100	0	0	56.17
	41	0	76.81	100	100
	42	100	63.24	100	100
	43	69.22	50.45	100	100
	48	100	100	100	100
	70	0	100	100	50.87
**RCC tumor** **—** **HPD (*n* = 10)**	55	100	100	100	100
	56	100	100	100	100
	57	100	100	0	100
	58	69.22	100	0	0
	59	0	84.30	100	100
	60	0	91.06	100	100
	61	0	0	100	78.48
	62	100	100	63.22	100
	63	100	100	80.40	100
	64	100	100	100	69.22
**RCC normal tissue—non-HPD (*n* = 12)**	90	0	0	0	34.77
	92	0	36.49	0	29.49
	97	0	0	43.57	29.49
	98	0	0	0	0
	99	0	0	20.24	34.92
	100	0	100	0	36.45
	101	19.90	0	0	0
	102	0	100	29.49	0
	103	0	100	28.73	35.51
	107	0	0	0	0
	110	0	0	0	50.69
	112	0	0	50.87	44.37
**RCC normal tissue** **—** **HPD (*n* = 5)**	85	0	0	0	0
	86	21.27	40.82	0	32.92
	87	0	100	37.09	0
	88	100	0	0	0
	89	0	0	0	0

Methylation percentages were determined by methylation-specific real-time PCR using melting curve peak height fluorescence values. Statistically significant differences (independent-samples *t*-test): *PTEN* (*p* = 0.005) and TP53 (*p* = 0.028) between MM HPD and MM non-HPD groups. Statistically significant differences (paired-samples *t*-test): PIK3CA (*p* < 0.001) and PTEN (*p* = 0.024) between tumor and matched normal tissue in the RCC non-HPD group. No significant gene-level differences were observed between RCC HPD and RCC non-HPD tumor samples, nor between tumor and normal tissue in the RCC HPD group. Abbreviations: HPD—hyperprogressive disease; MM—malignant melanoma; RCC—renal cell carcinoma.

**Table 2:** Somatic Variants Detected by Targeted Next-generation Sequencing in HPD Patients.

Patient ID	Diagnosis	TNM	Gene	Variant	dbSNP	AMP Tier	Pathogenicity	AF (%)
**49**	MM	T4bN0M1	*KIT*	NM_000222.3:c.1924A>G (p.Lys642Glu)	rs121913512	II	LP	43
**50**	MM	T4bN0M1	*PTEN*	NM_000314.8:c.988A>G (p.Lys330Glu)	rs1554825607	I	P	48
**51**	MM	T4bN1M1a	*BRCA2*	NM_000059.4:c.8881G>A (p.Gly2961Ser)	rs878853614	III	VOUS	—
**53**	MM	T4bN0M1	*MUTYH*	NM_001048174.2:c.1366G>A (p.Ala456Thr)	rs1441591597	III	VOUS	—
**53**	MM	T4bN0M1	*MUTYH*	NM_001048174.2:c.487C>T (p.Arg163Trp)	rs761101420	III	VOUS	—
**53**	MM	T4bN0M1	*CDH1*	NM_004360.5:c.1214A>G (p.Asn405Ser)	rs587778175	III	VOUS	—
**54**	MM	T4bN1M1c	*FANCA*	NM_000135.4:c.2567T>C (p.Leu856Ser)	rs370085403	III	VOUS	—
**54**	MM	T4bN1M1c	*FANCA*	NM_000135.4:c.1967C>T (p.Ala656Val)	—	III	VOUS	—
**55**	RCC	T2bN1M1	*—*	No pathogenic variants detected	—	—	—	—
**57**	RCC	T1aN0M1	*VHL*	NM_000551.4:c.344A>C (p.His115Pro)	—	I	LP	26
**59**	RCC	T2bN1M1	*KIT*	NM_000222.3:c.1924A>C (p.Lys642Gln)	rs121913512	I	P	38
**59**	RCC	T2bN1M1	*PALB2*	NM_024675.4:c.3306C>G (p.Ser1102Arg)	rs515726112	III	VOUS	—
**61**	RCC	T2bN0M1	*VHL*	NM_000551.4:c.332G>A (p.Ser111Asn)	rs869025631	I	P	29

Allele fractions are reported for pathogenic and likely pathogenic variants only. Abbreviations: AF—allele fraction; AMP—Association for Molecular Pathology; HPD—hyperprogressive disease; LP—likely pathogenic; MM—malignant melanoma; P—pathogenic; RCC—renal cell carcinoma; TNM—tumor–node–metastasis staging; VOUS—variant of uncertain significance. Variants were classified according to AMP/ASCO/CAP consensus recommendations [15].

## Data Availability

The data supporting the findings of this study are available from the corresponding author upon reasonable request.

## Supplementary Materials

The following supporting information can be downloaded at: https://www.mdpi.com/article/10.3390/jcm15135089/s1, Figure S1: MethPrimer output showing CpG island locations and methylation-specific primer positions for *PIK3CA* (A), *BAP1* (B), *PTEN* (C), and *TP53* (D); Figure S2: OncoPrint analysis of genes evaluated in the present study within (A) the TCGA Skin Cutaneous Melanoma (SKCM) and (B) the TCGA Kidney Renal Clear Cell Carcinoma (KIRC) cohort; Table S1: Comparison of methylation-included and non-included samples and patients; Table S2: Comparison of HPD-positive patients included and not included in NGS analysis; Table S3: Primer and amplicon details for methylation-specific real-time PCR assays; Table S4: Distribution of methylation percentage values obtained by MS-RT-PCR; Table S5: Targeted genes involved in cancer panel (86 genes).
