# Supplementary material for: Promoter Methylation and Somatic Mutations in Cancer-Related Genes Are Associated with Hyperprogressive Disease in Patients with Malignant Melanoma and Renal Cell Carcinoma Receiving Anti-PD-1/PD-L1 Immunotherapy
Source: J Clin Med. 2026 Jun 30;15(13):5089. doi: 10.3390/jcm15135089 (PMC13362896; doi:10.3390/jcm15135089)
Supplement: Supplementary file 1 [file jcm-15-05089-s001.zip › jcm-4262994-supplementary.pdf]

Supplementary Data

Supplementary Table S1. Comparison of methylation-included and non-included samples and patients.

Variable*	Included group	Non-included group	Total
**Sample number, n**	**54**	**59**	**113**
Tumor tissue samples, n	37	41	78
Normal tissue samples, n	17	12	29
Metastatic tissue samples, n	0	6	6
**Patient number, n**	**43**	**36**	**79**
HPD-positive cases among patients with known HPD status, n/N (%)	16/40 (40.0%)	1/34 (2.9%)	17/74 (22.9%)
Male, n/N (%)	35/43 (81.4%)	21/36 (58.3%)	56/79 (70.9%)
Female, n/N (%)	8/43 (18.6%)	15/36 (41.7%)	23/79 (29.1%)
Age, median	61 years	66 years	63 years
RCC diagnosis, n/N (%)	28/43 (65.1%)	17/36 (47.2%)	45/79 (57.0%)
MM diagnosis, n/N (%)	15/43 (34.9%)	19/36 (52.8%)	34/79 (43.0%)
Metastatic tissue available, n/N (%)	15/42 (35.7%)	14/36 (38.9%)	29/78 (37.2%)
Normal tissue available, n/N (%)	19/43 (44.2%)	10/36 (27.8%)	29/79 (36.7%)

* Sample-level variables include sample number and tissue type. Patient-level variables include sex, age, diagnosis, HPD status, and tissue availability. HPD status was available for 74 of 79 patients. Metastatic tissue availability was available for 78 of 79 patients. Abbreviations: HPD, hyperprogressive disease; MM, malignant melanoma; RCC, renal cell carcinoma.

Supplementary Table S2. Comparison of HPD-positive patients included and not included in NGS analysis.

Variable	NGS-included HPD group	Non-included HPD group	Total HPD-positive group
Patient number, n	9	8	17
NGS-analyzed tumor samples, n	9	0	9
Male, n/N (%)	9/9 (100.0%)	4/8 (50.0%)	13/17 (76.5%)
Female, n/N (%)	0/9 (0.0%)	4/8 (50.0%)	4/17 (23.5%)
Age*, median	70.5 years, n = 6	57 years, n = 7	65 years, n = 13
RCC diagnosis, n/N (%)	4/9 (44.4%)	7/8 (87.5%)	11/17 (64.7%)
MM diagnosis, n/N (%)	5/9 (55.6%)	1/8 (12.5%)	6/17 (35.3%)
Tumor tissue available, n/N (%)	9/9 (100.0%)	7/8 (87.5%)	16/17 (94.1%)
Normal tissue available, n/N (%)	2/9 (22.2%)	3/8 (37.5%)	5/17 (29.4%)
Metastatic tissue available**, n/N (%)	0/8 (0.0%)	6/8 (75.0%)	6/16 (37.5%)

* Age was unavailable for 4 of 17 HPD-positive patients.
** Metastatic tissue availability was available for 16 of 17 HPD-positive patients.
Abbreviations: HPD, hyperprogressive disease; MM, malignant melanoma; NGS, next-generation sequencing; RCC, renal cell carcinoma.

Supplementary Table S3. Primer and amplicon details for methylation-specific real-time PCR assays.

Gene	Reference sequence	Primer type	Forward primer	Reverse primer	Product size	Tm	MethPrimer start positions
PIK3CA	NC_000003.12	Methylated	TTTAATTGTATATAAATTTCGGGCG	AAACAAATACGCAAAAAAACGAA	128 bp	64.5°C	F: 118; R: 245
PIK3CA	NC_000003.12	Unmethylated	TTAATTGTATATAAATTTTGGGTGG	AAAACAAATACACAAAAAAACAAA	128 bp	62.6°C	F: 119; R: 246
BAP1	NC_000003.12	Methylated	GTAGTTATTTTCGGGCGGTC	GTTAACGATCGATTTCTACTAACGAT	139 bp	74.8°C	F: 79; R: 217
BAP1	NC_000003.12	Unmethylated	GTGGTAGTTATTTTTGGGTGGTT	ACCATTAACAATCAATTTCTACTAACAAT	145 bp	69.9°C	F: 76; R: 220
PTEN	NC_000010.11	Methylated	TTATAATAGTTTATTTTGTTTTCGG	AACCTACAACTACACTTACTACGAC	111 bp	73.3°C	F: 466; R: 576
PTEN	NC_000010.11	Unmethylated	TATAATAGTTTATTTTGTTTTTGG	AACCTACAACTACACTTACTACAAC	110 bp	69.7°C	F: 467; R: 576
TP53	NC_000017.11	Methylated	GCGTTTTTCGTTAAGATAGAAGC	ACGTAAAAAATTAAAAAATTTCCCG	100 bp	64.2°C	F: 215; R: 314
TP53	NC_000017.11	Unmethylated	AGGTGTTTTTTGTTAAGATAGAAGTGT	ATAAAAAATTAAAAAATTTCCCATT	100 bp	63.7°C	F: 213; R: 312

Primer sequences, product sizes, melting temperatures, and start positions were obtained from MethPrimer software. Start positions indicate MethPrimer-reported positions within the analyzed CpG island sequence and should not be interpreted as genomic chr:start-end coordinates. Methylated and unmethylated primer pairs were designed separately for each gene. Abbreviations: F, forward primer; R, reverse primer; Tm, melting temperature.

Supplementary Table S4. Distribution of methylation percentage values obtained by MS-RT-PCR.

Gene	0% values, n	Intermediate values, n	100% values, n	Total, n
PIK3CA	29	7	18	54
BAP1	24	9	21	54
PTEN	20	13	21	54
TP53	17	17	20	54

In the MS-RT-PCR workflow, 0% indicates detection of only the unmethylated-specific signal for the target region, whereas 100% indicates detection of only the methylated-specific signal. Intermediate values indicate detection of both methylated and unmethylated signals and were calculated from melting-curve peak-height fluorescence values using the standard curve.

Supplementary Table S5. Targeted genes involved in cancer panel (86 genes).

MTOR	*ARID1A*	*MPL*	*JAK1*	*NRAS*	*NTRK1*	*DDR2*	*DNMT3A*	*ALK*	*MSH2*	*MSH6*
NFE2L2	*IDH1*	*VHL*	*RAF1*	*MLH1*	*CTNNB1*	*SETD2*	*ATR*	*PIK3CA*	*FGFR3*	*PDGFRA*
FBXW7	*TERT*	*APC*	*RAD50*	*CSF1R*	*NPM1*	*ROS1*	*ESR1*	*PMS2*	*EGFR*	*MET*
BRAF	*EZH2*	*FGFR1*	*JAK2*	*CDKN2A*	*GNAQ*	*NTRK2*	*PTCH1*	*ABL1*	*TSC1*	*NOTCH1*
PTEN	*FGFR2*	*HRAS*	*ATM*	*KRAS*	*ERBB3*	*CDK4*	*PTPN11*	*HNF1A*	*POLE*	*FLT3*
RB1	*AKT1*	*MAP2K1*	*NTRK3*	*IDH2*	*TSC2*	*ERCC4*	*CDH1*	*FANCA*	*TP53*	*NF1*
ERBB2	*BRCA1*	*RAD51C*	*BRIP1*	*SMAD4*	*STK11*	*GNA11*	*MAP2K2*	*JAK3*	*ERCC2*	*SRC*
SMARCB1	*NF2*	*ERCC3*	*KIT*	*SMO*	*RET*	*BRCA2*	*RAD51D*	*GNAS*		

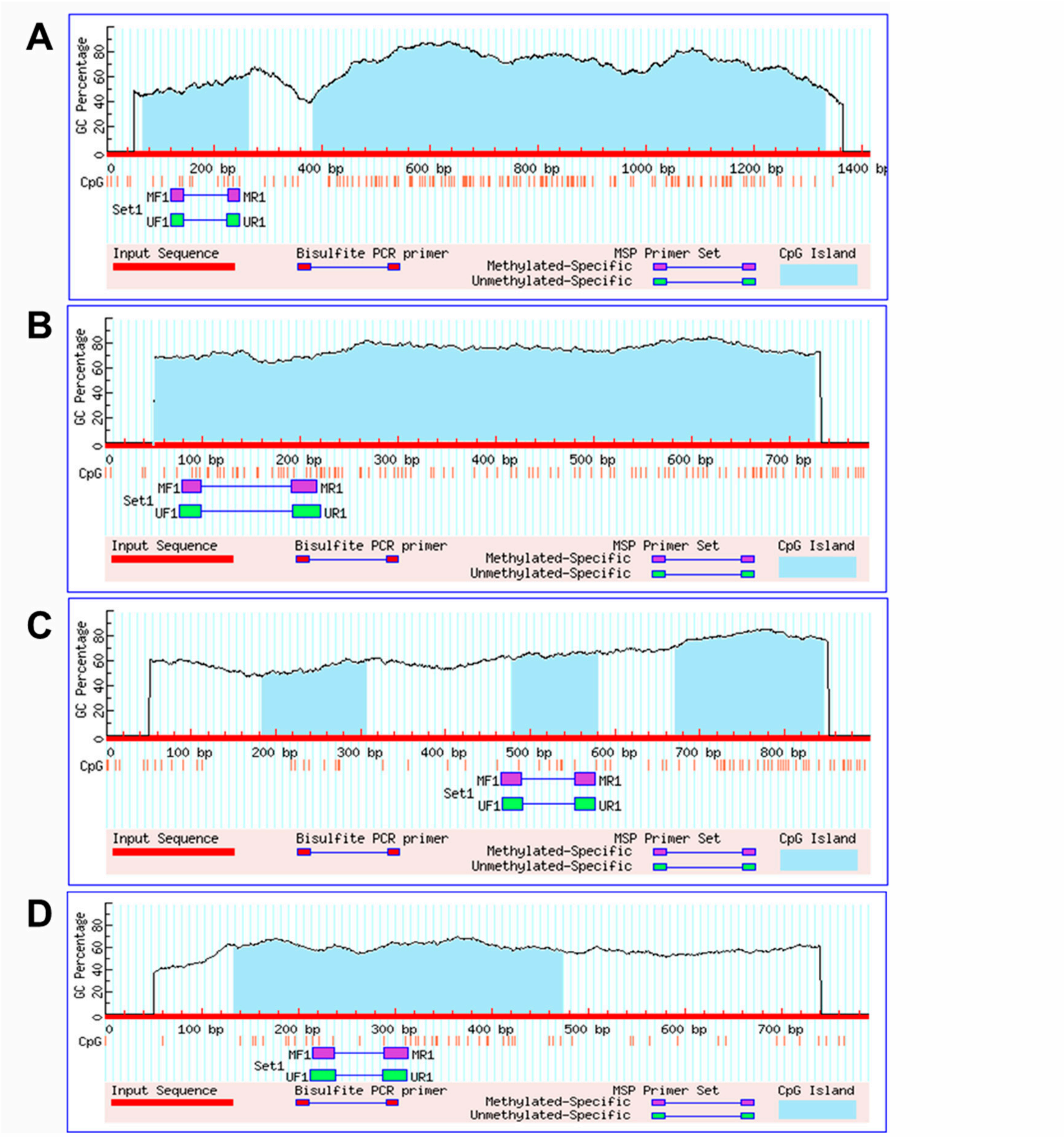

Supplementary Figure S1. MethPrimer output showing CpG island locations and methylation-specific primer positions for *PIK3CA* (A), *BAP1* (B), *PTEN* (C), and *TP53* (D).

A

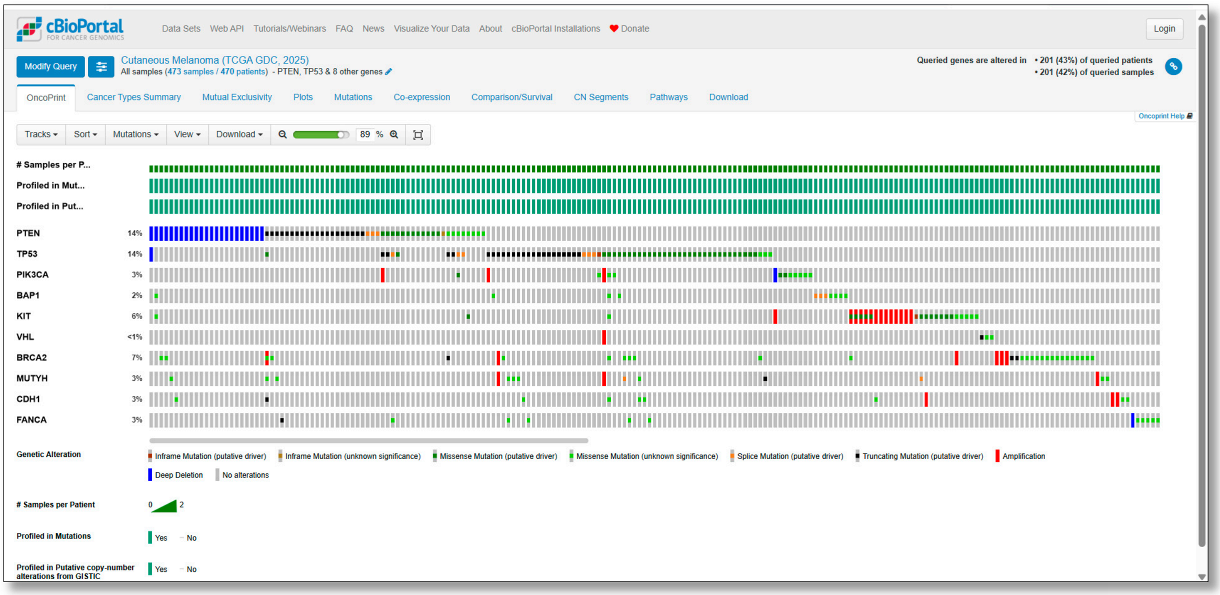

B

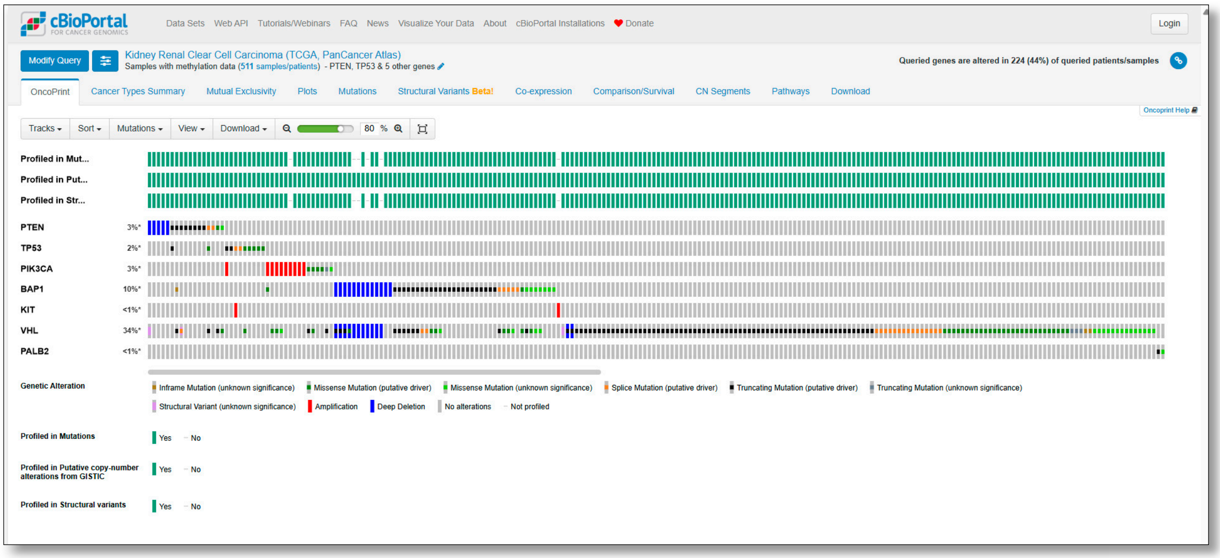

Supplementary Figure S2. OncoPrint analysis of genes evaluated in the present study within (A) the TCGA Skin Cutaneous Melanoma (SKCM) and (B) the TCGA Kidney Renal Clear Cell Carcinoma (KIRC) cohort.
